# Creatine target engagement with brain bioenergetics: a dose-ranging phosphorus-31 magnetic resonance spectroscopy study of adolescent females with SSRI-resistant depression

**DOI:** 10.1007/s00726-016-2194-3

**Published:** 2016-02-23

**Authors:** Douglas G. Kondo, Lauren N. Forrest, Xianfeng Shi, Young-Hoon Sung, Tracy L. Hellem, Rebekah S. Huber, Perry F. Renshaw

**Affiliations:** 1 Department of Psychiatry, University of Utah School of Medicine, 501 Chipeta Way, Salt Lake City, UT 84108 USA; 2 The Brain Institute, 383 Colorow Drive, Salt Lake City, UT 84108 USA

**Keywords:** Creatine monohydrate, Phosphocreatine, Bioenergetics, Mitochondrial function, Adolescent depression, Women’s health, Phosphorus-31 magnetic resonance spectroscopy

## Abstract

Major depressive disorder (MDD) often begins during adolescence and is projected to become the leading cause of global disease burden by the year 2030. Yet, approximately 40 % of depressed adolescents fail to respond to standard antidepressant treatment with a selective serotonin reuptake inhibitor (SSRI). Converging evidence suggests that depression is related to brain mitochondrial dysfunction. Our previous studies of MDD in adult and adolescent females suggest that augmentation of SSRI pharmacotherapy with creatine monohydrate (CM) may improve MDD outcomes. Neuroimaging with phosphorus-31 magnetic resonance spectroscopy (^31^P-MRS) can measure the high-energy phosphorus metabolites in vivo that reflect mitochondrial function. These include phosphocreatine (PCr), a substrate for the creatine kinase reaction that produces adenosine triphosphate. As part of the National Institute of Mental Health’s experimental medicine initiative, we conducted a placebo-controlled dose-ranging study of adjunctive CM for adolescent females with SSRI-resistant MDD. Participants were randomized to receive placebo or CM 2, 4 or 10 g daily for 8 weeks. Pre- and post-treatment ^31^P-MRS scans were used to measure frontal lobe PCr, to assess CM’s target engagement with cerebral energy metabolism. Mean frontal lobe PCr increased by 4.6, 4.1 and 9.1 % in the 2, 4 and 10 g groups, respectively; in the placebo group, PCr fell by 0.7 %. There was no group difference in adverse events, weight gain or serum creatinine. Regression analysis of PCr and depression scores across the entire sample showed that frontal lobe PCr was inversely correlated with depression scores (*p* = 0.02). These results suggest that CM achieves target engagement with brain bioenergetics and that the target is correlated with a clinical signal. Further study of CM as a treatment for adolescent females with SSRI-resistant MDD is warranted.

## Introduction

Depressive disorders are the second leading cause of years lived with disability as of 2010 (Ferrari et al. 2013) and major depressive disorder (MDD) is projected to become the leading cause of global disease burden by 2030 (Lepine and Briley 2011). The onset of MDD commonly occurs during adolescence (Burke et al. 1991); in fact, MDD has a cumulative prevalence of 15–24 % by age 19 (Lewinsohn et al. 1998; Merikangas et al. 2010) and results in significant disability and mortality in this age group (Birmaher et al. 2007). Selective serotonin reuptake inhibitors (SSRIs) are first-line medications for adolescents requiring MDD pharmacotherapy (Lewandowski et al. 2013); however, ~40 % of depressed adolescents fail to respond to initial treatment with an SSRI (Brent et al. 2008). These adolescents with treatment-resistant depression (TRD) are at risk for recurrence into adulthood, academic and social impairment and suicide (Zhou et al. 2014). The Treatment of SSRI-Resistant Depression in Adolescents (TORDIA) trial found that ~60 % of adolescents with TRD did not respond, when switched to a different SSRI or another antidepressant class (Brent et al. 2008). An alternative to switching medications is to introduce an adjunctive treatment, a strategy that has shown promise in the management of adults with TRD (Rush et al. 2009). Despite the public health importance of improving care for adolescent MDD, controlled clinical trials of augmentation strategies for adolescents with TRD are lacking.

During puberty (Angold et al. 1998), the prevalence of MDD among females rises to approximately twice that found in males (prevalence ratio 1.3–3.1; lifetime gender ratio 2.1) (Kuehner 2003), a finding that is robust to epidemiologic sampling across continents and cultures (Seedat et al. 2009). Thus, female adolescents are affected by MDD at twice the rate of males (Merikangas et al. 2010), a gender disparity that continues throughout the reproductive years (Bebbington et al. 2003; Robins and Regier 1991). Yet despite the evidence for gender-based differences in MDD pathophysiology (Hyde et al. 2008; Young and Korszun 2010) and treatment response (Yang et al. 2011; Bigos et al. 2009), little systematic research has focused on these empirical differences. In addition to the doubling of depression prevalence by the middle teenage years (Wade et al. 2002), girls with MDD have longer episodes and higher symptom severity compared to boys (McCauley et al. 1993) and a prolonged risk of recurrence compared with women whose first depressive episode occurs in adulthood (Kovacs 1996). Therefore, investigation of females with MDD during the critical ‘window of vulnerability’ that occurs in adolescence (Andersen and Teicher 2008; Hankin et al. 1998) provides a unique opportunity to reduce the burden of illness associated with depression.

Converging lines of evidence suggest that mitochondrial dysfunction and altered brain bioenergetics may contribute to the etiology of depression (Klinedinst and Regenold 2015; Gardner and Boles 2011). The neuroimaging technique phosphorus-31 magnetic resonance spectroscopy (^31^P-MRS) is the sole method for noninvasive, in vivo measurement of the high-energy phosphorus metabolites that reflect cerebral bioenergetics (Agarwal et al. 2010; Kondo et al. 2011a; Zhu et al. 2009). These include phosphocreatine (PCr), beta-nucleoside triphosphate (β-NTP; largely adenosine triphosphate, ATP) and inorganic phosphate (Pi) (Iosifescu et al. 2008). PCr is the substrate reservoir for the creatine kinase reaction (Wallimann et al. 1992; Jeong et al. 2011). In mitochondria, this reaction reversibly converts PCr into ATP + creatine in a 1:1 molar ratio (Brosnan and Brosnan 2007; Wallimann et al. 2011). Neuronal energy demands are met through a shift in reaction equilibrium, which is designed to maintain brain ATP concentrations constant (Andres et al. 2008; Schlattner et al. 2006).

Our previous translational studies of MDD reveal a pattern in baseline/pre-treatment ^31^P-MRS metabolites, in subjects who later respond to treatment in a clinical trial setting: increased PCr (Iosifescu et al. 2008) and decreased β-NTP (Renshaw et al. 2001). The pattern is more common in females (Renshaw et al. 2001); this fact, when combined with the evidence for gender dimorphism in frontal lobe PCr and Pi in healthy females (Riehemann et al. 1999), suggested that it might be possible to target bioenergetic metabolism in the development of novel treatments for females with MDD. To investigate this, we first sought to determine whether the concentration of ^31^P-MRS metabolites in brain could be modified by administration of creatine monohydrate (CM). In a placebo-controlled study of healthy adults, we found that cerebral PCr and Pi increased and β-NTP decreased, in subjects receiving oral CM versus placebo (Lyoo et al. 2003). This reproduced the alterations in ^31^P-MRS metabolites that predict responsiveness, in two distinct groups of MDD patients: (1) treatment-naïve subjects receiving initial pharmacotherapy with an SSRI (Renshaw et al. 2001); and (2) SSRI-resistant subjects who receive hormonal augmentation of their existing antidepressant (Iosifescu et al. 2008). These results raised the possibility that CM supplementation could be utilized to promote a ‘treatment-responsive state’ in both MDD and TRD. As an initial step, we recruited unmedicated female adults with MDD for a placebo-controlled trial of CM versus placebo, each added to the SSRI escitalopram. Compared with the SSRI + placebo group, subjects who received SSRI + creatine showed greater improvements in depression scores as early as week 2 of treatment (Lyoo et al. 2012), suggesting that adjunctive CM speeds response and increases remission in women with MDD. An open-label study of TRD in either MDD or BD also found that CM showed evidence of benefit in unipolar depression (Roitman et al. 2007). In parallel with our study of adult females, we also conducted a small-scale (*n* = 5) open-label study of CM in adolescent females with TRD who had failed to respond to a trial of the SSRI fluoxetine. ^31^P-MRS scans of the frontal lobe were acquired at baseline and repeated after 8 weeks of CM 4 g daily, with healthy control (HC) adolescent females scanned for comparison. The study’s results included a mean 56 % reduction in CDRS-R depression scores and an increase in PCr (*p* = 0.02) in female adolescents with TRD compared to HC (Kondo et al. 2011b).

In an effort to accelerate treatment development in psychiatry, the U.S. National Institute of Mental Health (NIMH) recently announced the NIMH Experimental Medicine Initiative (Insel 2015). The initiative requires that NIMH-funded clinical trials shift their emphasis from tests of clinical efficacy, e.g., reduction in depression scores, to studies of disease mechanism (Insel and Gogtay 2014). This requires a translational demonstration of *target engagement*, where the ‘target’ is a molecular site or brain circuit linked to a mechanism of disease, the mechanism of action of the treatment under study, or both. The new trial format is designed to yield insight into how or for whom the targeted intervention works and potentially measures duration and dose–response. The data from clinical trials are then informative, regardless of a study’s results: if the intervention engages the target and correlates with a signal in the clinical data, then development of the treatment—and those aimed at the same target—continues. If not, then the target’s mechanistic relevance is ruled out, and the focus shifts to identifying new treatment targets. Based upon the preliminary studies described above, we received one in the initial wave of NIMH phased innovation awards (R21/R33). This report presents the findings from the R21 proof-of-principle study, a dose-ranging trial of CM for adolescent females with SSRI-resistant depression. The CM dose administered in our open-label study was 4 g daily, which was safe and well tolerated (Kondo et al. 2011b). We therefore tested one lower dose (2 g) and one higher dose (10 g) in this study, in addition to placebo. The specified target of CM treatment was frontal lobe PCr, because of its association with treatment response in prior studies of MDD and TRD. In fact, pre-treatment PCr predicts subsequent treatment ‘responder’ versus ‘non-responder’ status in TRD with an accuracy of 79 % (83 % sensitivity; 75 % specificity) (Iosifescu et al. 2008). Because our open-label adolescent data were collected in females, there were no extant placebo-controlled CM data in males with TRD and, perhaps, most importantly, because preclinical animal data suggest CM has antidepressant-like effects in female rats but is equivocal in males (Allen et al. 2010, 2015), the study’s enrollment was limited to adolescent females. In addition, more recent data suggest CM’s antidepressant-like effects are sex hormone dependent (Allen et al. 2015). The aims of this R21 were: to demonstrate that CM targets and alters brain energy metabolism in female adolescents with SSRI-resistant MDD in a dose-responsive manner, compared with placebo; and to determine the dose of CM offering the best combination of safety, tolerability and target engagement with mitochondrial function in these participants.

## Methods

This was a single-site, placebo-controlled, dose-finding clinical trial of CM augmentation for adolescent females with SSRI-resistant MDD (ClinicalTrials.gov registry number: NCT01601210). The study was conducted under U.S. Food and Drug Administration Investigational New Drug (IND) application #104586 issued to Dr. Renshaw, and the University of Utah Institutional Review Board (IRB) approved this research. Written and verbal informed consent was obtained prior to performance of any research-related procedures: (1) parental permission and participant assent, for participants aged <18 years; or (2) participant consent, for participants ≥ age 18 years. An external Data Safety and Monitoring Board with authority to halt the study monitored the study’s safety outcomes and risks to participants.

### Participants

Participants were recruited through clinician referrals and IRB-approved advertising. The inclusion criteria were: females aged 13–20 years with a primary diagnosis of MDD; outpatient status; SSRI treatment for ≥8 weeks at a dosage of ≥40 mg/day fluoxetine or its equivalent; current Children’s Depression Rating Scale-Revised (CDRS-R) (Poznanski and Mokros 1996; Mayes et al. 2010) raw score of >40 or Montgomery–Asberg Depression Rating Scale (MADRS) (Montgomery and Asberg 1979) score >25; and current Clinical Global Impression Scale-Severity (CGI-S) (Guy 1976) score ≥4, indicating moderate severity of the current depressive episode. Diagnoses were established through administration of the Kiddie Schedule for Affective Disorders and Schizophrenia for School-Age Children-Present and Lifetime Version (K-SADS-PL) (Kaufman et al. 1997), or the Structured Clinical Interview for DSM-IV Axis I Disorders (SCID-CT) (First et al. 2007). All psychopharmacological treatments were stable for ≥30 days at study entry, and participants were asked to hold their psychotropic medication regimens constant during the 8 weeks of randomized treatment. The study exclusion criteria were: pre-existing renal disease or proteinuria on baseline urinalysis testing; history of CM hypersensitivity or a previous failed therapeutic trial of CM; concomitant treatment with antiepileptic or antipsychotic medications; current substance use disorder; unstable comorbid medical, neurological or psychiatric disorder; clinically significant suicidal or homicidal risk; positive pregnancy test or refusal to practice contraception or abstinence during the study; current breastfeeding; positive urine drug screen; known or suspected intellectual disability; and any contraindication to ^31^P-MRS imaging, e.g. ferromagnetic implant or claustrophobic anxiety. A complete blood count, comprehensive metabolic panel, urinalysis, urine drug screen and 12-lead electrocardiogram (ECG) were obtained at baseline, and testing was repeated at the conclusion of randomized treatment, to prospectively identify abnormalities associated with CM administration. Participants attended weekly clinic visits for the first 4 weeks and then bi-weekly visits through week 8 of treatment. At each visit, the CDRS-R and CGI-S were administered.

### Phosphorus-31 magnetic resonance spectroscopy (^31^P-MRS) neuroimaging

Neuroimaging was performed on a Siemens 3 T whole-body clinical scanner (Siemens Medical Solutions, Erlangen, Germany) that is FDA approved for clinical use. Participants first underwent a routine MRI protocol to acquire anatomic images in the axial and coronal planes. The protocol consists of a T1-weighted structural scan (MPRAGE), a double-echo T2-weighted scan and a fluid attenuated inversion recovery (FLAIR) scan. Examinations were performed with a quadrature radiofrequency coil (Clinical MR Solutions LLC, Brookfield, WI, USA). After localization, images were obtained using a T1-weighted, sagittal oriented 3D-magnetization prepared rapid gradient echo (MPRAGE) sequence (TR/TE/TI 2100/3.97/1100 ms, matrix size 256 × 256, FOV 256 × 256 mm^3^, flip angle 12°, slice thickness 1.5 mm, slab 192 mm, bandwidth 190 Hz/pixel). Axial proton density and T2-weighted images were acquired to screen for structural abnormalities using 2D double-echo T2-weighted turbo spin echo (TSE) sequence (TR 7110 ms, TE 28/84 ms, FOV 240 × 210 mm, slice thickness 3 mm, flip 150°, bandwidth 179 Hz/pixel). A FLAIR sequence (TR/TE/TI 8000/90/2500 ms, slice thickness 5 mm, FOV 240 × 168 mm^3^, voxel size 0.8 × 0.6 × 5.0 mm, bandwidth 200 Hz/pixel, turbo factor 13) was used to detect juxtacortical–cortical lesions. All anatomic MRI images were read by the University of Utah faculty radiologists.

**Fig. 1:**
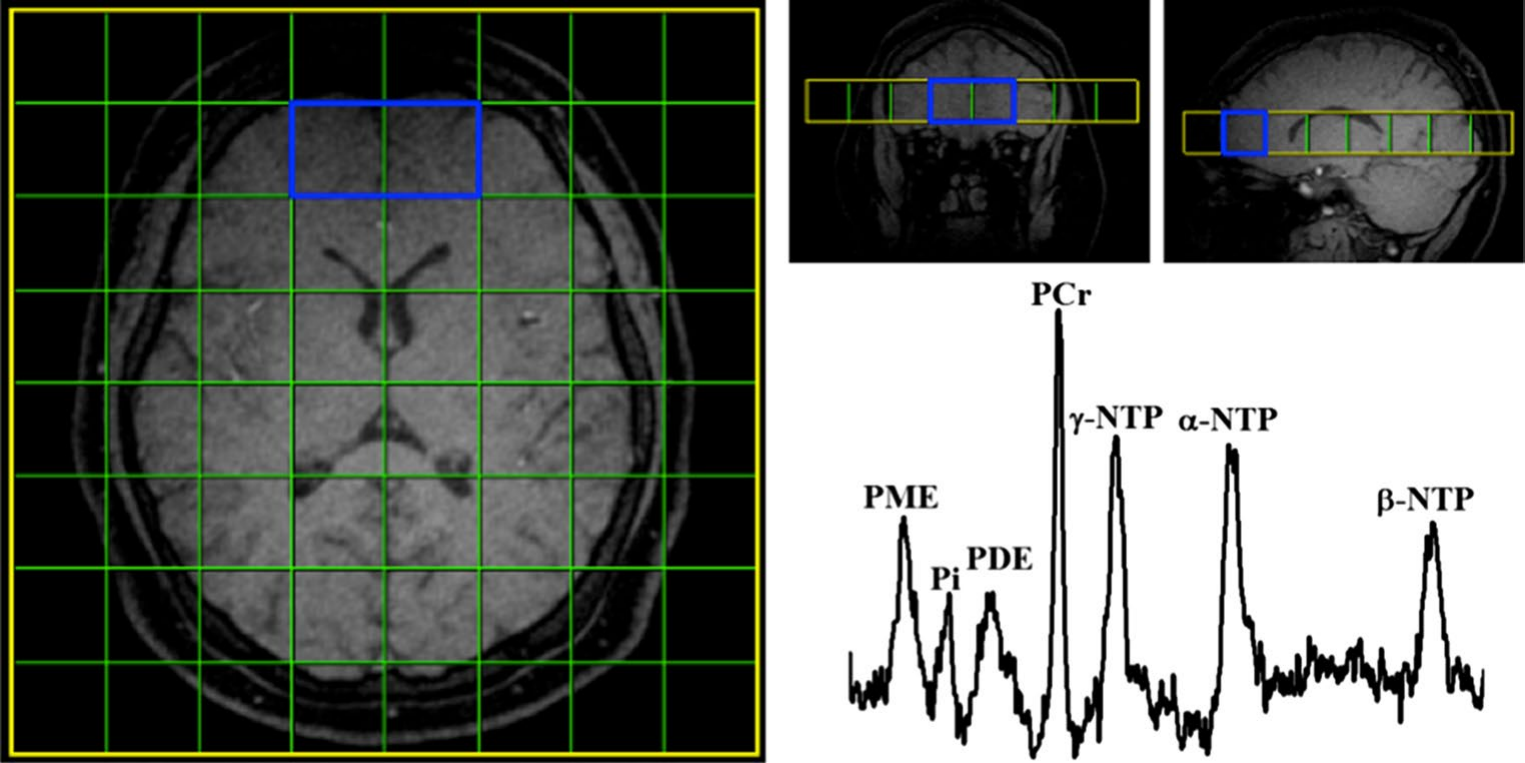
Frontal lobe region of interest for phosphorus-31 magnetic resonance spectroscopy scans and a representative 31P-MRS spectrum. *NTP* alpha, gamma and beta-nucleoside triphosphate, *PCr* phosphocreatine, *PDE* phosphodiesters, *Pi* inorganic phosphate, *PME* phosphomonoesters

^31^P-MRS data were acquired using a 3D-MRS sequence with elliptically weighted phase encoding, to minimize acquisition time, i.e., participant burden. The acquisition parameters were: data matrix size 8 × 8 × 8, TR 2000 ms, flip angle 90° for hard RF pulse, Rx bandwidth 2.5 kHz, complex points 1024, readout duration 409 ms, pre-acquisition delay 2.3 ms, FOV 200 × 200 mm^2^ and 16 number of excitations. Within the Siemens operator interface, the RF pulse-transmitted voltage was varied until the maximal phosphorus signal intensity within the volume of interest (VOI) was achieved. That voltage was deemed to be generating a 90° flip angle. However due to field inhomogeneity, the true RF flip angle may vary across different brain tissues. Therefore to minimize the effects of RF field inhomogeneity, ^31^P-MRS results are expressed as the ratio of the individual metabolite concentrations to the total phosphate signal acquired from the VOI. Post-processing of ^31^P-MRS data was performed with the jMRUI software application (jMRUI v. 4.0, European Community) with the AMARES algorithm (Advanced Method for Accurate, Robust and Efficient Spectral fitting of MRS data with use of prior knowledge). Before fitting the free-induction-decay (FID) data, a Hamming filter was applied to reduce signal contamination from neighboring voxels, with apodization of 10 Hz line broadening. Fourier transformation, frequency shifts correction and zero-order/first order phase correction as well as baseline correction were applied. The structural image-processing tool FSL (FMRIB Software Library, Release 4.1, The University of Oxford) was used to estimate the gray matter (GM), white matter (WM) and cerebrospinal fluid (CSF) content of each voxel, to correct for the partial volume effects on the metabolite data. The MRS grid was positioned over the images in identical fashion for both scans. For each ^31^P-MRS metabolite, the peak area was calculated as a percentage of the total phosphorus (TP) signal acquired during the participant’s scan. Figure 1 shows the region of interest in the frontal lobe slice and a representative ^31^P-MRS spectrum.

### Statistical analysis

Group differences in demographic and baseline/pre-treatment variables among the four treatment groups were assessed using the analysis of variance (ANOVA) procedure. The linear mixed model (LMM) procedure was utilized for analysis of the primary outcome measure, frontal lobe ^31^P-MRS PCr, and the secondary outcome measure, CDRS-R depression scores (Bland and Altman 1994; Roy 2006; Cnaan et al. 1997). Because the neuroimaging and clinical measures were correlated, repeated measures data, a robust estimate of the variance, was obtained by implementing the vce(robust) option (Huber 1967; White 1980) in the statistical software Stata^®^ for Linux, release 13 (StataCorp LP, College Station, TX, USA).

## Results

**Fig. 2:**
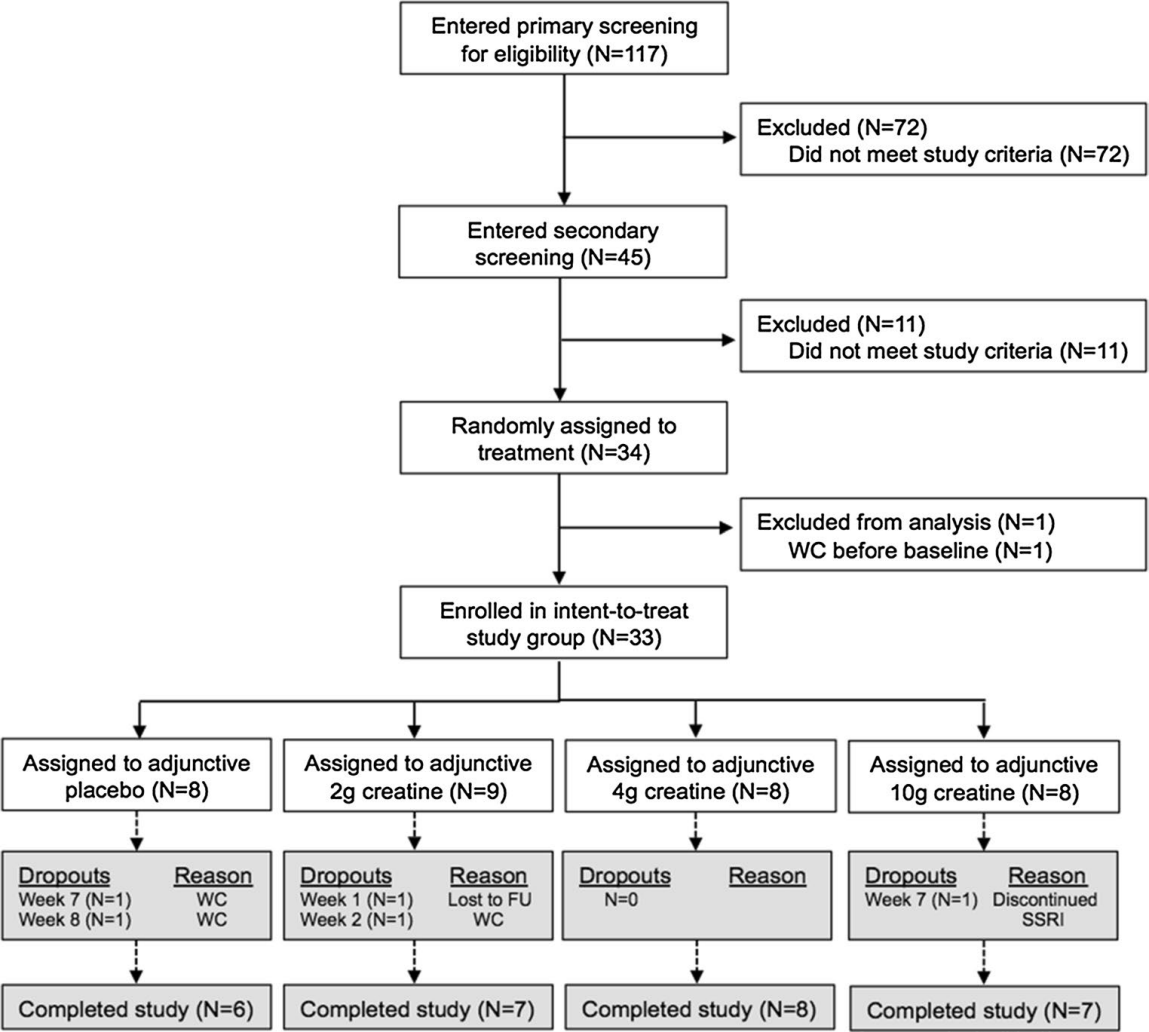
Participant flow diagram: screening, randomization and disposition of adolescent females with treatment-resistant major depressive disorder. *FU* follow-up, *SSRI* selective serotonin reuptake inhibitor, *WC* withdrew consent

As shown in Table 1 and displayed in the flow diagram in Fig. 2, we enrolled a total of *n* = 34 participants. *N* = 45 participants were formally assessed using the K-SADS-PL or SCID-CT, of which *n* = 11 did not meet the diagnostic inclusion criteria. One participant met the criteria, but relocated 300 miles from Salt Lake City and, therefore, withdrew from further participation prior to her baseline/pre-treatment scan. Complete data including two ^31^P-MRS scans were available for *n* = 28 participants. Because the primary outcome measure of target engagement was frontal lobe PCr, statistical analyses were performed on the data collected from these ‘protocol completers’. The sample included one African-American participant and two participants who self-identified as Hispanic or Latino.

**Table 1:** Participant baseline characteristics (*N* = 34)

	N	%	p*
*Demographic variables*			
Race			
White	33	97.1	0.39
African-American	1	2.9	
Ethnicity			
Not Hispanic or Latino	32	94.1	0.60
Hispanic or Latino	2	5.9	
Handedness			
Right	30	88.2	0.60
Left	1	2.9	
Missing	3	8.8	

	Mean	SD	
Age (years)	17.1	2.09	0.73
Clinical variables			
Comorbid conditions	1.33	0.85	.046
Clinical Rating Scale Scores			
CDRS-R	57.52	6.29	0.48
MADRS	26.85	5.68	0.14
CGI-S	4.42	0.56	0.40

	*N*	%	
SSRI treatment			0.29
Fluoxetine	9	27.3	
Citalopram	6	18.2	
Escitalopram	4	12.1	
Sertraline	14	42.4	
Concomitant medications			0.48
Mixed amphetamine salts	1	2.9	
Mirtazapine	1	2.9	
Oral contraceptive	2	5.9	
Bupropion	2	5.9	
Vitamin D	1	2.9	
Dropout	5	15.0	0.52

*CDRS-R* Children’s Depression Rating Scale-Revised, *CGI-S* Clinical Global Impressions-Severity, *C-SSRS* Columbia-Suicide Severity Rating Scale, *MADRS* Montgomery–Asberg Depression Rating Scale

* ANOVA

Table 2 includes the baseline and week 8 outcomes, for the protocol completers who were randomized to each of four treatment conditions. The results of the ANOVA procedure indicated that there was no significant difference between groups at baseline, in terms of age (*p* = 0.38), pre-treatment frontal lobe PCr (*p* = 0.75) or CDRS-R raw score at study entry (*p* = 0.51).

**Table 2:** Protocol completer outcome measures: means and standard deviations

Variable	Placebo	Creatine 2 g	Creatine 4 g	Creatine 10 g	*p* value anova
Number of participants	*N* = 6	*N* = 7	*N* = 8	*N* = 7	–
Mean age (years)	16.8 (1.06)	17.6 (1.86)	16.4 (2.63)	17.0 (2.61)	0.38
PCr/TP (*N* = 28)					
Frontal lobe, baseline	0.149 (0.005)	0.151 (0.006)	0.145 (0.005)	0.143 (0.005)	0.75
Frontal lobe, final	0.148 (0.004)	0.158 (0.004)	0.151 (0.003)	0.156 (0.003)	0.32
Change (%)	−0.7	+4.6	+4.1	+9.1	0.69
β-NTP/TP (*N* = 28)					
Frontal lobe, baseline	0.111 (0.015)	0.106 (0.013)	0.109 (0.011)	0.100 (0.021)	0.55
Frontal lobe, final	0.104 (0.008)	0.106 (0.015)	0.099 (0.001)	0.103 (0.012)	0.86
Change (%)	−6.3	0	−9.1	+3	0.47
PME/TP (*N* = 28)					
Frontal lobe, baseline	0.145 (0.012)	0.134 (0.020)	0.138 (0.011)	0.140 (0.009)	0.53
Frontal lobe, final	0.132 (0.011)	0.138 (0.013)	0.145 (0.014)	0.143 (0.008)	0.28
Change (%)	−9.0	3.0	5.1	2.1	0.72
PDE/TP (*N* = 28)					
Frontal lobe, baseline	0.163 (0.022)	0.167 (0.017)	0.169 (0.007)	0.167 (0.019)	0.93
Frontal lobe, final	0.169 (0.007)	0.159 (0.018)	0.171 (0.020)	0.163 (0.011)	0.54
Change (%)	3.7	−4.8	1.2	2.4	0.60
CDRS-R mean raw score					
Baseline	58.2 (3.96)	62.5 (9.35)	58.0 (8.53)	56.9 (5.05)	0.51
Final	43.0 (12.58)	34.8 (8.20)	41.8 (14.78)	36.1 (13.96)	0.59
Change (%)	−26	−44	−29	−37	–
Participants reporting Gastrointestinal adverse events	*n* = 4/6	*n* = 2/7	*n* = 5/8	*n* = 4/7	–
Weight gain					
Pounds	2.83 (3.65)	2.90 (2.39)	2.33 (8.54)	5.28 (3.80)	0.75
Percent	+1.8	+2.2	+1.7	+3.9	0.64
Serum creatinine					
Baseline (mg/dL)	0.78 (0.056)	0.85 (0.066)	0.65 (0.056)	0.77 (0.056)	0.16
Final (mg/dL)	0.79 (0.051)	0.84 (0.062)	0.74 (0.057)	0.88 (0.053)	0.32

*CDRS-R* Children’s Depression Rating Scale, Revised, *TP* total phosphorus signal, *PCr* phosphocreatine, *β-NTP* beta-nucleoside triphosphate, *PME* phosphomonoester, *PDE* phosphodiester

In terms of adverse events, gastrointestinal side effects were most commonly reported by participants in the placebo arm (*n* = 4 of 6; 67 %). Participants experienced mild weight gain in all treatment conditions including placebo, as measured either in pounds (range 2.33–5.28 pounds; *p* = 0.75) or in percent gained (range 1.7–3.9 %; *p* = 0.64). Regarding safety, questions have been raised in the literature regarding CM’s potential to impair renal function. Accordingly, participants’ serum creatinine levels were measured prior to administration of study drug and repeated following 8 weeks of randomized treatment. We found no significant group difference in participants’ mean serum creatinine, either at baseline (range 0.65–0.85 mg/dL; *p* = 0.16) or at week 8 (range 0.74–0.88 mg/dL). In terms of the tolerability and acceptability of adjunctive CM, no subject withdrew from the study due to CM-associated adverse events. Participants lost to follow-up included two who were unable to tolerate the baseline ^31^P-MRS scan and elected not to repeat it and the participant who relocated. No participant attempted suicide or was psychiatrically hospitalized, and there were no serious adverse events, as defined by the FDA. There were no unresolved treatment-emergent adverse events, abnormalities on laboratory testing or 12-lead ECGs.

**Fig. 3:**
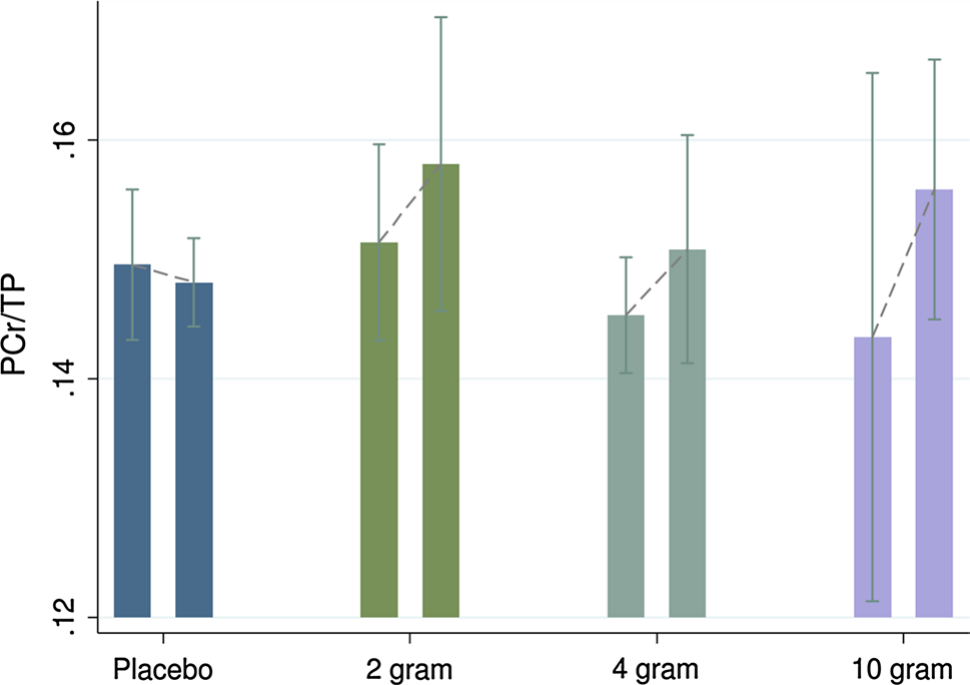
Pre- versus post-treatment frontal lobe phosphocreatine (PCr/TP) in participants randomized to treatment with placebo or creatine 2, 4 or 10 g daily (*N* = 28)

The results of ^31^P-MRS showed a tendency for frontal lobe PCr to increase by 4.6, 4.1 and 9.1 % in the CM 2, 4 and 10 g groups, respectively (Fig. 3; *p* = 0.69). By contrast in the placebo group, frontal lobe PCr displayed a tendency to decrease by 0.7 %; frontal lobe PCr changes across all four treatment groups did not achieve statistical significance (Table 2; *p* = 0.69). We also found that only the 10 g CM group demonstrated an increase in frontal lobe β-NTP: from baseline to week 8, the observed difference in β-NTP was −6.3 % in the placebo group, 0 % in the 2 g CM group, −9.1 % in the 4 g CM group and +3 % in the 10 g CM group (*p* = 0.47).

**Fig. 4:**
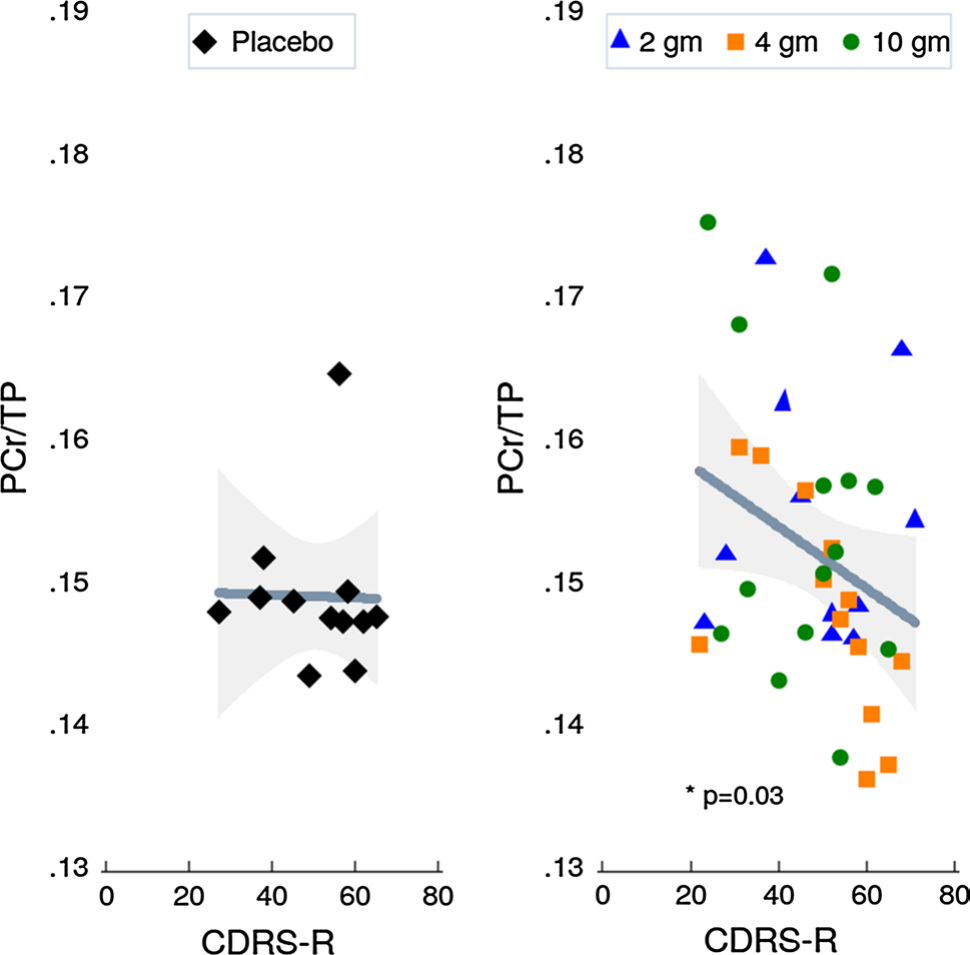
Correlation between Children’s Depression Rating Scale-Revised (CDRS-R) scores and frontal lobe phosphocreatine (PCr/TP) and in participants randomized to placebo (*left*) versus active treatment (*right*) with creatine 2, 4 or 10 g (*p = 0.03)

**Fig. 5:**
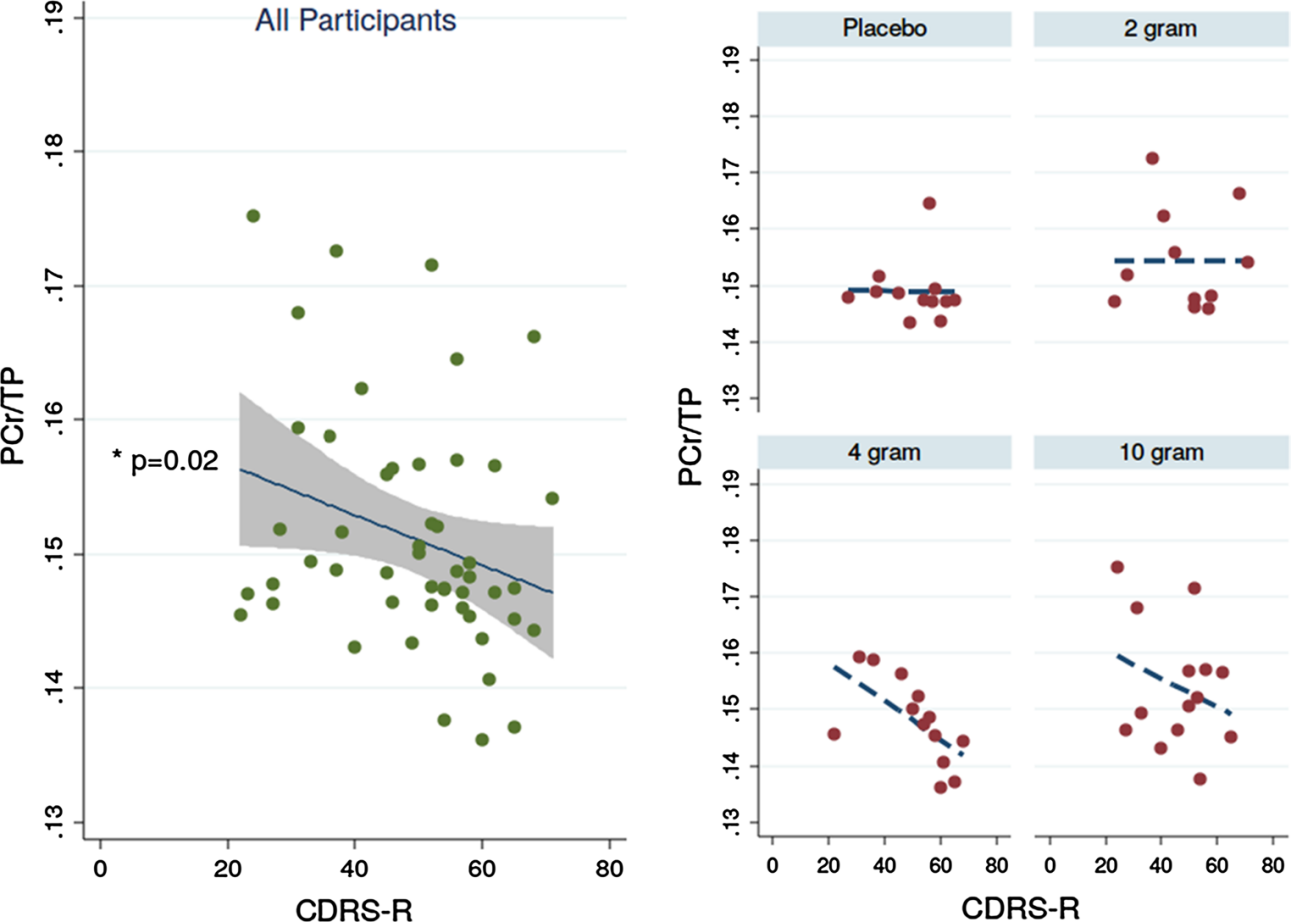
Correlation between Children’s Depression Rating Scale-Revised (CDRS-R) scores and frontal lobe phosphocreatine (PCr/TP) and in the entire sample (*p* = 0.02). *Right panel* scatter plot of the correlation between Children’s Depression Rating Scale-Revised (CDRS-R) scores and frontal lobe PCr/TP, displayed by treatment group: placebo, 2 g creatine, 4 g creatine and 10 g creatine. *Left panel* negative correlation between changes in Children’s Depression Rating Scale-Revised (CDRS-R) scores and frontal lobe phosphocreatine/total phosphorus signal (PCr/TP) across all phosphorus magnetic resonance spectroscopy scans, i.e., lower depression scores correlate with higher PCr/TP concentrations (*p = 0.02)

However as shown in Fig. 4, when the three active CM treatment groups were combined (*n* = 25) and compared with the placebo group (*n* = 8) utilizing an LMM analysis of frontal lobe PCr versus CDRS-R scores, there was a significant negative correlation in the CM-treated group, i.e., higher frontal lobe PCr correlates with lower depression scale scores (*p* = 0.03); this relationship was not present in the placebo group. Furthermore, when the four treatment groups are combined and all frontal lobe PCr measurements are regressed versus all CDRS-R depression scores recorded at study scan visits, the negative correlation between frontal lobe PCr and CDRS-R scores becomes marginally more significant (*p* = 0.02), as shown in the left panel of Fig. 5. The right panel of Fig. 5 displays the individual scatter plots of the placebo, CM 2, 4 and 10 g groups. Visual inspection of the regression lines suggests that the 4 and 10 g treatment groups were responsible for the majority of the variance in the relationship between frontal lobe PCr and CDRS-R score. ^31^P-MRS can also be used to measure local concentrations of phosphomonoesters (PME) and phosphodiesters (PDE). PME consist of phospholipid membrane precursors, while PDE are phospholipid breakdown products. Although we found no significant between-group differences in post-treatment PME, visual inspection of the raw data shows that PME/TP decreased by 9 % in the placebo group, while PME/TP was marginally increased in the three active CM treatment groups. While no conclusion can be drawn from this observation, it is perhaps notable that vesicular uptake of glutamate, a neurotransmitter implicated in mood disorders, may be regulated by PME and PCr (Xu et al. 1997). The results of the analysis of the mean percent tissue content of the VOI among the four treatment groups were as follows: GM pre-treatment (*p* = 0.32), GM post-treatment (*p* = 0.73), WM pre-treatment (*p* = 0.15), WM post-treatment (*p* = 0.79), CSF pre-treatment (*p* = 0.82), CSF post-treatment (*p* = 0.92).

**Fig. 6:**
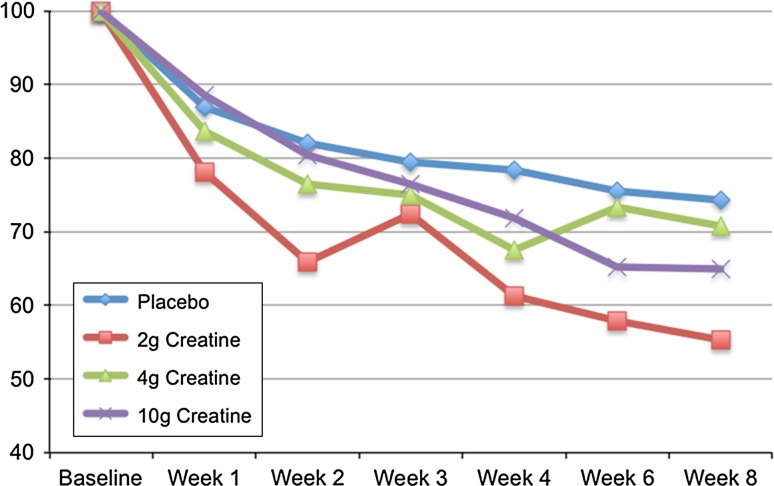
Percent Change in Children’s Depression Rating Scale-Revised (CDRS-R) scores from baseline to week 8, displayed by treatment group (*N* = 28)

Finally, Fig. 6 displays the percent change in CDRS-R clinical depression scores by treatment group, over the 8 weeks of randomized treatment. We did not find a statistically significant between-group difference in CDRS-R scores at week 8 (*p* = 0.59), nor does the change in depression score appear to be dose-dependent. This may be related to the study sample size and the fact that participants randomized to the 2 g group had the highest mean CDRS-R score at baseline. Among the four groups, participants in the 2 g daily (mean CDRS-R = 34.8) and 10 g daily (mean CDRS-R = 36.1) groups no longer met the study’s depression severity inclusion criterion (CDRS-R > 40) at the end of treatment.

## Discussion

The authors report the results of what is, to the best of our knowledge, the first ^31^P-MRS experimental medicine dose-ranging study of adjunctive CM for female adolescents with SSRI-resistant MDD. While our results diverge with those of a recent dose-ranging study of CM augmentation in adults with TRD (Nemets and Levine 2013), the outcome of interest in the present report was PCr measured with translational ^31^P-MRS neuroimaging. As found in our previous open-label study (Kondo et al. 2011b), augmentation of SSRI pharmacotherapy with CM was associated with increased frontal lobe PCr. Open treatment with 4 g CM daily was associated with a 6.4 % increase in the prior trial. In the present study, adjunctive 2, 4 and 10 g of CM daily for a total of 8 weeks was associated with tendencies toward increased frontal lobe PCr of 4.6, 4.1 and 9.1 %, respectively. By comparison, participants in the placebo group demonstrated a tendency toward *decreased* frontal lobe PCr of 0.7 %. In addition, target engagement with frontal lobe PCr was correlated with scores on the standard measure of depression severity employed in child psychiatry clinical trials, the CDRS-R (Figs. 3, 4). In terms of outcomes, participants randomized to the 2 g dose of CM appeared to show a tendency toward the most robust clinical response, as measured by CDRS-R scores (Fig. 6); however, this was not statistically significant (*p* = 0.59). This squares with what little is known regarding the response to placebo vs. active pharmacotherapy in adolescents with TRD. Zhou et al. (2014) performed a systematic review of adolescent TRD treatments and found that the only randomized, placebo-controlled trial in this population found no difference in the response rate to amitriptyline (76.9 %) versus placebo (78.6 %) (Birmaher et al. 1998). The largest study of adolescent TRD conducted to date is the TORDIA clinical trial, which did not include a placebo arm among its four treatment conditions (Brent et al. 2008). Meanwhile, the NIMH has adopted the *experimental medicine* model for clinical research, moving away from tests of clinical efficacy to focus on disease mechanisms (Insel and Gogtay 2014; Insel 2015). The present dose-ranging study found that CM 10 g achieves target engagement with PCr, while concomitant increases in adverse events such as gastrointestinal symptoms, weight gain or increased serum creatinine were not observed. Adding to the rationale for selecting the 10 g dose is the fact that pre-treatment PCr is an accurate predictor of treatment response in TRD (Iosifescu et al. 2008), and creatine’s neuroprotective effect on brain bioenergetics is dose dependent, occurring at higher dosages (Atassi et al. 2010). Thus the relevance of the target, and its engagement by CM, appeared to be confirmed, and development of adjunctive CM as a treatment for TRD in adolescent females will continue. We have opened recruitment on the next phase of development, an NIMH-sponsored R33 placebo-controlled pilot study of CM 10 g for female adolescents with SSRI-resistant MDD, the results of which can be used to inform the design and implementation of a larger-scale efficacy study.

In addition to PCr, another measure of mitochondrial function often reported in ^31^P-MRS studies of MDD is β-NTP, which is considered a proxy measure for the concentration of ATP. We did not find a discernible tendency in alterations in frontal lobe β-NTP in this study. However, there are important differences in the study samples, participant characteristics and VOI, between previous reports and the present study. For example, we previously reported that in healthy adult males, administration of CM for 14 days was associated with a significant decrease in the β-NTP resonance (*p* = 0.026) obtained from axial brain slice through the orbitofrontal and occipital cortices (Lyoo et al. 2003). We also found an 18 % decrease in basal ganglia β-NTP (*p* < 0.03), in unmedicated adults with MDD versus healthy volunteers (Moore et al. 1997). This was followed by our report that basal ganglia β-NTP is 21 % lower in MDD treatment responders versus non-responders (*p* = 0.02) to SSRI fluoxetine (Renshaw et al. 2001). Another group reported decreased β-NTP in the frontal lobe (*p* = 0.001), in a sample of *n* = 14 adult men and women with MDD compared with *n* = 10 healthy volunteers (Volz et al. 1998). The distinctions between these findings and the current report include the adolescent age group, an all-female sample and the fact that all of our participants were not only medicated, but treatment resistant at baseline, i.e., “non-responders.” When added to the fact that the earlier studies were performed on 1.5 T scanning systems, these systematic differences in (1) magnet strength; (2) gender; (3) age range; (4) depression severity; (5) medication status; and (6) VOI, could plausibly account for the difference in findings with respect to β-NTP. Furthermore in their seminal ^31^P-MRS report, Kato et al. (1992) found no significant differences in β-NTP concentration among the following groups: MDD depressed, MDD euthymic, bipolar depressed, bipolar euthymic and healthy controls. In contrast, however, PCr was significantly decreased in severe versus mild MDD, and there was a non-significant tendency toward decreased PCr in bipolar depressed versus bipolar euthymic subjects (Kato et al. 1992).

MDD is a chronic, recurrent illness that frequently has its origins in adolescence (Burke et al. 1991). Unfortunately, the current antidepressants appear to possess limited efficacy in juvenile depression (Tsapakis et al. 2008), and adolescent MDD (Avenevoli et al. 2015) and TRD (Zhou et al. 2014) remain substantial public health concerns. The scope of the problem is highlighted by the fact that 350 million individuals suffer from depression worldwide (World Health Organization 2015). Depression costs are estimated at $200 billion annually in the USA (Mrazek et al. 2014), surpassing the total spent on cancer or diabetes mellitus (Ionescu et al. 2015). Because the burden of MDD falls disproportionately on girls and women, successful development of interventions for female adolescents in this critical period of development could have a broad impact on public health.

The mitochondrial dysfunction associated with depression (Gardner and Boles 2011; Klinedinst and Regenold 2015) presents a tractable target for developing novel therapeutics and may also offer opportunities to improve the efficiency of drug development (Manji et al. 2012). In the context of the NIMH Experimental Medicine Initiative (Insel 2015), ^31^P-MRS is a translational tool capable of measuring ‘target engagement’ by quantifying the high-energy phosphorus-bearing neurometabolites involved in brain bioenergetics (Iosifescu and Renshaw 2003; Kato et al. 1992). One of these, PCr, has been shown to predict treatment response in patients who have not responded to antidepressant pharmacotherapy (Iosifescu et al. 2008) and thus present a rational treatment target in TRD. This study joins prior reports from our group (Lyoo et al. 2003; Hellem et al. 2015; Kondo et al. 2011b) and others (Pan and Takahashi 2007; Bianchi et al. 2007) in providing in vivo evidence that oral CM administration achieves target engagement with brain bioenergetics and the creatine kinase system.

Numerous directions for further study of CM suggest themselves. For example, all protocol completers in the present study were offered a 6-month open-label extension of CM 4 g daily, the dose administered in our open-label study. The rationale for this is twofold. Foremost is the obligation the investigators feel to offer participants randomized to placebo an opportunity to benefit from CM augmentation. Additionally, it is important to assess the durability of treatment effects and to collect long-term safety and tolerability data. The results of the 6-month extension study will be reported separately. Mechanistically, there is still much to be learned regarding CM’s downstream effects in the brains of depressed individuals. A series of elegant experiments from the Rodrigues laboratory point toward an overlap in the neurobiological pathways involved in the antidepressant-like effect of CM in animal models and those of ketamine (Cunha et al. 2014, 2015a, b, c). It is perhaps notable that ketamine is considered a ‘rapid’ antidepressant, and in our study of adult females with MDD, CM appeared to accelerate the antidepressant effects of the SSRI escitalopram (Lyoo et al. 2012). Based upon the gender dimorphism of creatine’s effects in our rodent studies (Allen et al. 2010, 2012), there is the intriguing possibility of studying CM’s antidepressant effect as monotherapy for untreated female adolescents with MDD. Given that antidepressants increase the risk of suicidal behavior in depressed youth (Hammad et al. 2006), this strategy may be worth exploring. Finally, in consideration of the converging evidence for sex-based differences in brain mitochondrial functioning (Demarest and McCarthy 2015), there may also be opportunities to explore gender-specific pharmacotherapies in depression. We have taken an initial step in that direction, with a study of CM supplementation to target depressive symptomatology in females with substance use disorders (Hellem et al. 2015). The gender dimorphism in rodent creatine kinase activity in brain has led investigators to conclude that females are more dependent on the system, between sexual maturity and senescence (Ramirez and Jimenez 2002). Recent data indicate that gender, sex hormones and metabolic status mediate the behavioral and neurochemical effects of CM supplementation. Specifically, combined CM and sex hormone treatment normalizes neuroplasticity-related gene expression in gonadectomized rats of both sexes and has an antidepressant-like effect in females (Allen et al. 2015). Given these findings and the evidence for sexual dimorphism in how brain mitochondria respond to both stress-depression paradigms and to subsequent treatment with SSRI medications (Adzic et al. 2013), further investigation of antidepressant strategies targeting brain bioenergetics in females with MDD is warranted.

This study’s findings should be interpreted in light of several limitations. Chief among these is its small sample size, which limited our power to detect differences in the primary and secondary outcome measures between our four treatment groups. Two studies of adolescent depression implementing interventions other than CM, the TADS (March et al. 2004) and TORDIA (Brent et al. 2008) clinical trials, randomized *n* = 439 and *n* = 334 participants, respectively, to four different treatment groups. Furthermore, dose-ranging studies that employ conventional clinical outcomes must enroll > 100 participants per dosing regimen tested (Erondu et al. 2006). Sample sizes of that magnitude are not feasible for translational neuroimaging studies and point toward the rationale for transitioning to ‘target engagement’ as the primary outcome measure for human subject research in the NIMH Experimental Medicine Initiative (Potter 2015). Another limitation is the low rate of creatine transporter (SLC6A8) expression in human brain endothelium (Lowe et al. 2015), which supports the suggestion that there is modest permeability for creatine at the blood–brain barrier (Braissant et al. 2001) and that de novo synthesis in glia may serve as the principal source of creatine in brain (Braissant et al. 2007; Tachikawa et al. 2004). Following intraperitoneal injection of creatine to rats, no significant increase in either creatine or PCr can be measured (Perasso et al. 2003). A recent report used established methods to estimate dietary creatine intake (Harris et al. 1997) and concluded that that diet does not influence brain creatine content and that the human brain is dependent on its own creatine biosynthesis (Yazigi Solis et al. 2014). While our own clinical trials (Lyoo et al. 2003; Hellem et al. 2015; Kondo et al. 2011b) and those of other investigators (Dechent et al. 1999; Atassi et al. 2010; Turner et al. 2015; Pan and Takahashi 2007) support the idea that CM administration alters creatine and/or PCr in brain, it is a limitation that all of these studies used a single method—magnetic resonance spectroscopy—to measure subjects’ brain chemistry. A frequent limitation of neuroimaging studies in psychiatry is the potential for confounding due to psychotropic medications, and the present report is no exception. Previous reports have documented the potential for antidepressants to alter neuroimaging results in females with MDD (Kaymak et al. 2009) and in adolescents (Singh and Chang 2012). Thus, it must be acknowledged that SSRI administration may have added noise to our ^31^P-MRS acquisitions, for which the signal-to-noise ratio is a critical source of variance. A final limitation that restricts the generalizability of our findings is that we studied only females. Future studies of CM in male adolescents with TRD may be warranted, but for ethical reasons we will need to await completion of at least one trial demonstrating CM’s safety in adult males with depression.

In summary, we present the results of a double-blind, randomized, placebo-controlled dose-ranging clinical trial of adjunctive CM for female adolescents with SSRI-resistant MDD. Participants receiving 10 g appeared to show a greater degree of alteration in brain energy metabolism measured with repeated ^31^P-MRS neuroimaging, without concomitant differences in adverse events, tolerability or treatment acceptability. Furthermore, frontal lobe PCr, the putative target of CM administration, demonstrated a correlation with clinical measures of depression across our study sample. Our results join converging lines of evidence from multiple scientific disciplines suggesting that CM may provide benefit in a range of brain-based disorders (Rae and Broer 2015; Allen 2012). We have now opened recruitment on a two-arm, placebo-controlled trial of CM 10 g for female adolescents with TRD. The results of that study will inform Go/No Go decision making regarding further development of CM and provide insight into the utility of adopting the experimental medicine framework for the discovery and validation of novel treatments in psychiatry.
